# ERCC6L promotes cell growth and metastasis in gastric cancer through activating NF-κB signaling

**DOI:** 10.18632/aging.203387

**Published:** 2021-08-23

**Authors:** Dehu Chen, Qinghong Liu, Gan Cao

**Affiliations:** 1 Department of General Surgery, Taizhou People’s Hospital, The Fifth Affiliated Hospital of Nantong University, Taizhou 225300, China

**Keywords:** ERCC6L, gastric cancer, epithelial-mesenchymal transition, NF-κB

## Abstract

ERCC6L has been reported to act as a potential oncogenic protein in various cancers. However, the role of ERCC6L in the progression of gastric cancer (GC) remains to be elucidated. Herein, we aimed to assess the clinical significance, the role, and the underlying mechanism of ERCC6L in GC progression. In this study, the mRNA and protein expression levels of ERCC6L were measured in GC specimens by quantitative real-time PCR (qRT-PCR), Western blot, and immunohistochemistry, and its clinical significance was assessed. The effect of ERCC6L overexpression or knockdown on GC cell growth, migration, and invasion was explored by functional experiments. Notably, the possible mechanisms underlying the action of ERCC6L were also investigated. We found that ERCC6L was upregulated in GC tissues, and its expression was associated with tumor size, clinical stage, and poor prognosis in GC patients. Besides, ERCC6L facilitated GC cell proliferation and metastasis *in vitro* and *in vivo*. Mechanically, ERCC6L modulated GC cell behavior via activation of NF-κB signaling. Our results indicated that ERCC6L played a critical role in GC progression and metastasis. In addition, ERCC6L promoted GC cell growth and metastasis via activation of NF-κB signaling, thus possibly providing a target for GC.

## INTRODUCTION

Worldwide, gastric cancer (GC) is one of the most common malignant tumors [1–2]. Despite advances in the development of surgery and chemotherapy in recent years, the prognosis of GC patients remains dissatisfactory as a result of recurrence and metastasis rates [3]. Therefore, identifying novel predictive tumor biomarkers as well as understanding the mechanisms responsible for GC development may provide targets useful for improving GC outcomes.

Recently, growing evidence has revealed that epithelial-mesenchymal transition (EMT) is a fundamental process of tumor initiation and progression. During EMT, epithelial cells lose their intercellular junction, reprogram gene expression and undergo a substantial change in signalling programme; this switch modifies the cell shape to promote motility and invasion [4]. Molecularly, a hallmark of EMT is E-cadherin level downregulation and N-cadherin level upregulation. These changes in gene expression are regulated by transcription factors as well as an intricate network of signalling pathways, including TGF-β, JAK-STAT, PI3K/Akt, MAPK, and NF-κB [5].

Human ERCC6L is recognized as an embryonic development-related protein [6]. Recently, ERCC6L also appears to play an important role in cancer progression. As reported, ERCC6L upregulation was demonstrated to be correlated with tumor progression and poor prognosis in hepatocellular carcinoma (HCC) [7]. Additionally, ERCC6L was found to promote colorectal cancer (CRC) cell growth and invasion [8]. However, the pathophysiologic role and the mechanism of ERCC6L-induced GC cell growth and metastasis are still unclear.

In this study, we investigated the role and the potential molecular of ERCC6L in GC progression.

## RESULTS

### ERCC6L expression is elevated in GC patients and is closely correlated with poor clinical outcomes

**Figure 1:**
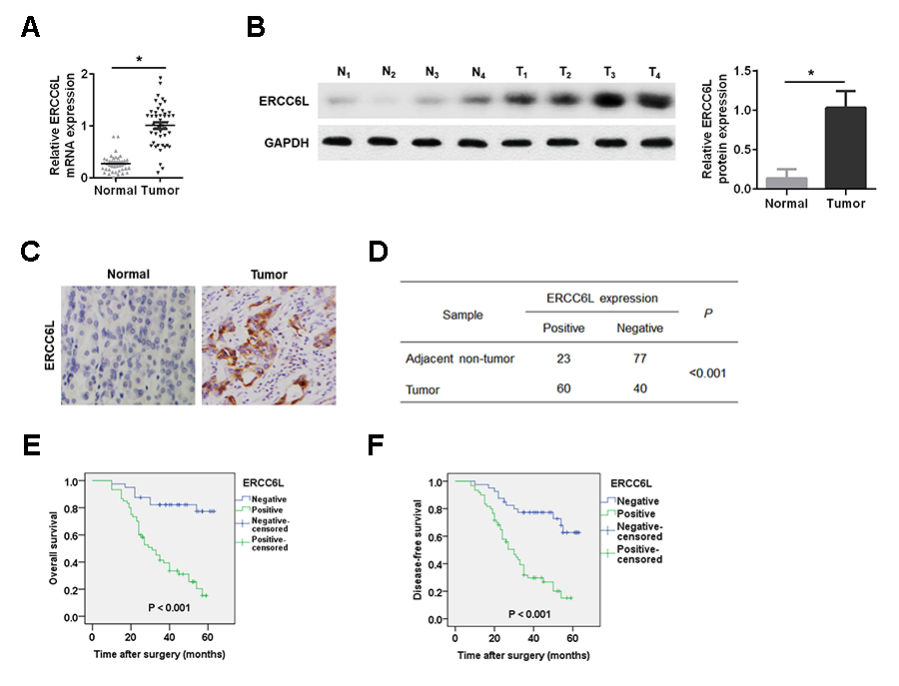
**ERCC6L expression in GC tissues.** (**A**) qRT-PCR detection of ERCC6L mRNA expression in GC tissues and adjacent normal tissues. (**B**) Western blot analysis of ERCC6L protein expression in tumor tissues (T) and matched normal tissues (N). (**C**) IHC analysis of ERCC6L expression in GC tissues and matched normal tissues. (**D**) Chi-square analysis of ERCC6L expression in GC tissues and adjacent non-tumor tissues based on IHC staining scores. (**E**) Kaplan-Meier analysis of overall survival for GC patients with different levels of ERCC6L expression. (**F**) Kaplan-Meier analysis of disease-free survival for GC patients with different levels of ERCC6L expression. ^*^P < 0.05.

We found that ERCC6L mRNA and protein expressions were evidently higher in cancer tissues compared with those in adjacent normal tissues in 45 pairs of cases (Figure 1A and 1B). Likewise, The IHC analysis indicated the similar result (Figure 1C and 1D).

Next, we found that high ERCC6L expression indicated poor clinical outcomes in GC patients (Table 1). Kaplan-Meier analysis turned out that high ERCC6L expression in GC patients led to poor overall survival (Figure 1E) and/or disease-free survival (Figure 1F).

**Table 1:** Relationship between ERCC6L expression and clinicopathological parameters in gastric cancer.

**Clinicopathological Parameters**	***n***	**ERCC6L**		***P*-value**
		**+**	**–**	
**Age (years)**				
≥60	69	41	28	0.860
<60	31	19	12	
**Gender**				
Male	72	41	31	0.317
Female	28	19	9	
**Tumor size (cm)**				
≥5	64	44	20	**0.017**
<5	36	16	20	
**Lauren’s classification**				
Diffuse	29	19	10	0.472
Intestinal	71	41	30	
**Lymphatic vessel invasion**				
With	38	24	14	0.614
Without	52	36	26	
**T stage**				
T_1_ + T_2_	45	19	26	**0.001**
T_3_ + T_4_	55	41	14	
**pTNM stage**				
I + II	39	16	23	**0.002**
III + IV	61	44	17	
**Lymph node metastasis**				
With (N_1_ + N_2_ + N_3_)	59	46	13	**<0.001**
Without (N_0_)	41	14	27	

### ERCC6L induces EMT in GC cells

**Figure 2:**
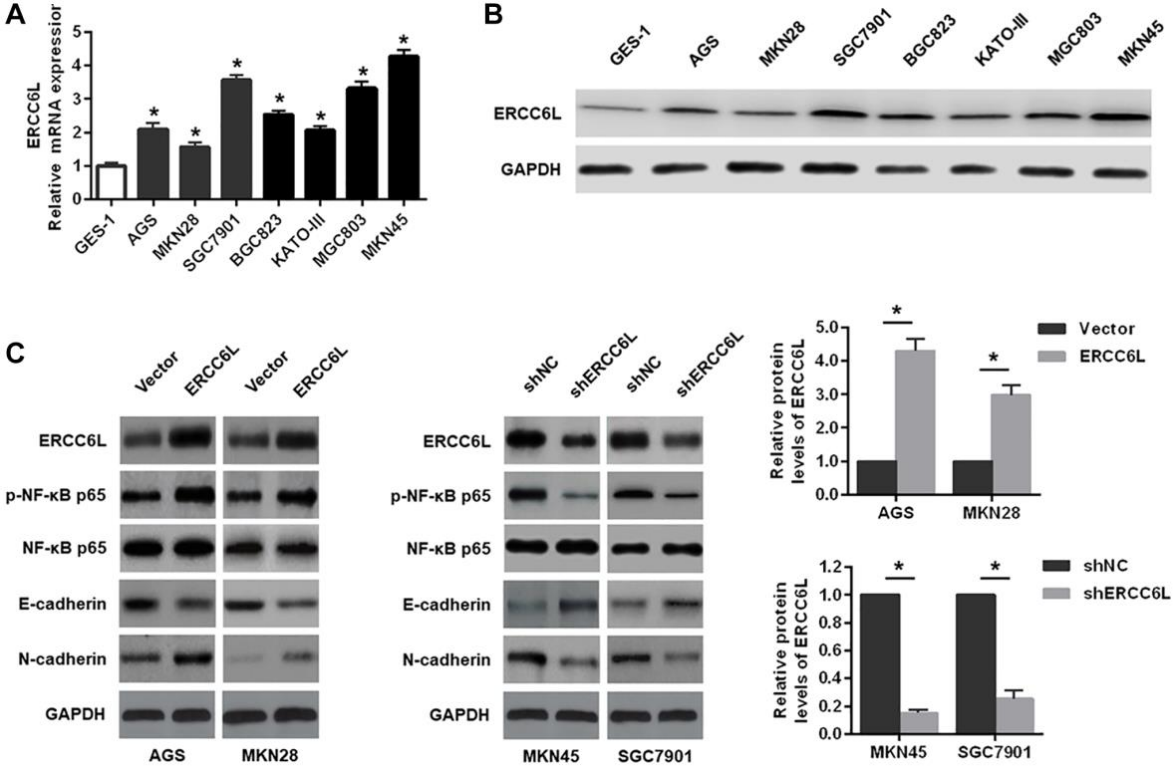
**The effects of ERCC6L overexpression or knockdown on the EMT and NF-κB signaling-related markers in GC cells.** (**A**) qRT-PCR detection of relative ERCC6L mRNA expression in human GC cell lines and a normal gastric epithelial cell line. (**B**) Western blot analysis of ERCC6L protein expression in human GC cell lines and a normal gastric epithelial cell line. (**C**) Western blot analysis of the effects of ERCC6L overexpression or knockdown on the EMT and NF-κB signaling-related markers in GC cells. ^*^P < 0.05.

Compared with GES-1, the mRNA and protein expressions of ERCC6L were significantly elevated in GC cells (AGS, MKN28, SGC7901, BGC823, KATO-III, MGC803 and MKN45) (Figure 2A and 2B). Subsequently, we found that ERCC6L overexpression increased p-NF-κB p65 and N-cadherin expression levels, and decreased E-cadherin expression in AGS cells and MKN28 cells. On the contrary, ERCC6L downregulation in MKN45 and SGC7901 cells led to the opposite outcomes (Figure 2C).

### ERCC6L promotes proliferation, migration and invasion of GC cells *in vitro*

**Figure 3:**
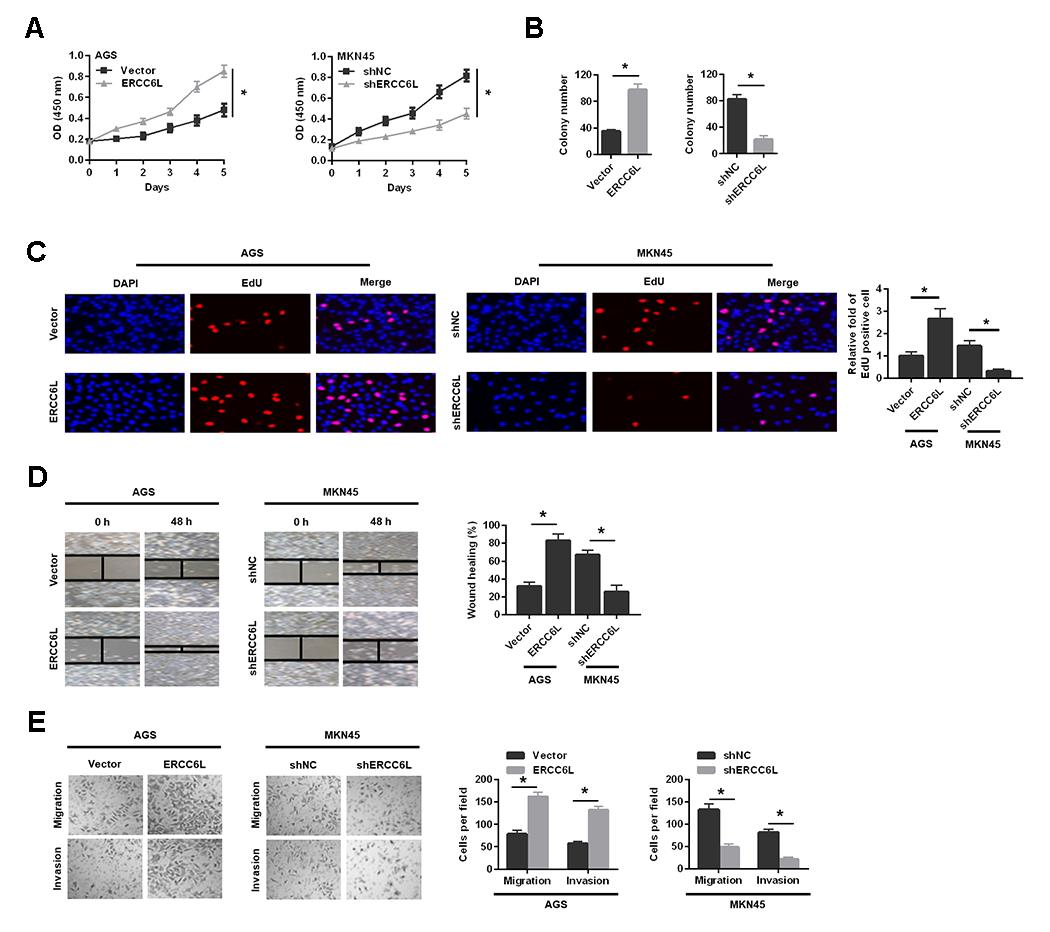
**The effects of ERCC6L overexpression or knockdown on GC cells growth, migration and invasion *in vitro*.** Following ERCC6L overexpression or knockdown treatment, CCK-8 assay (**A**), colony formation assay (**B**) and EdU assay (**C**) were used to evaluate the effects of ERCC6L expression on the proliferation ability of GC cells. The wound-healing assay (**D**), transwell migration and invasion assays (**E**) were employed to assess the effects of ERCC6L overexpression or knockdown on the migration and invasion ability of GC cells. ^*^P < 0.05.

We found that ERCC6L overexpression markedly promoted cell growth in AGS cells, while ERCC6L knockdown significantly suppressed cell growth in MKN45 cells (Figure 3A–3C). Additionally, we found that ERCC6L overexpression markedly promoted cell migration and invasion in AGS cells, whereas ERCC6L knockdown in MKN45 cells observably led to the opposite outcomes (Figure 3D and 3E). In summary, these data suggested that ERCC6L promoted proliferation, migration and invasion of GC cells *in vitro*.

### ERCC6L promotes tumor formation and metastatic potential *in vivo*

**Figure 4:**
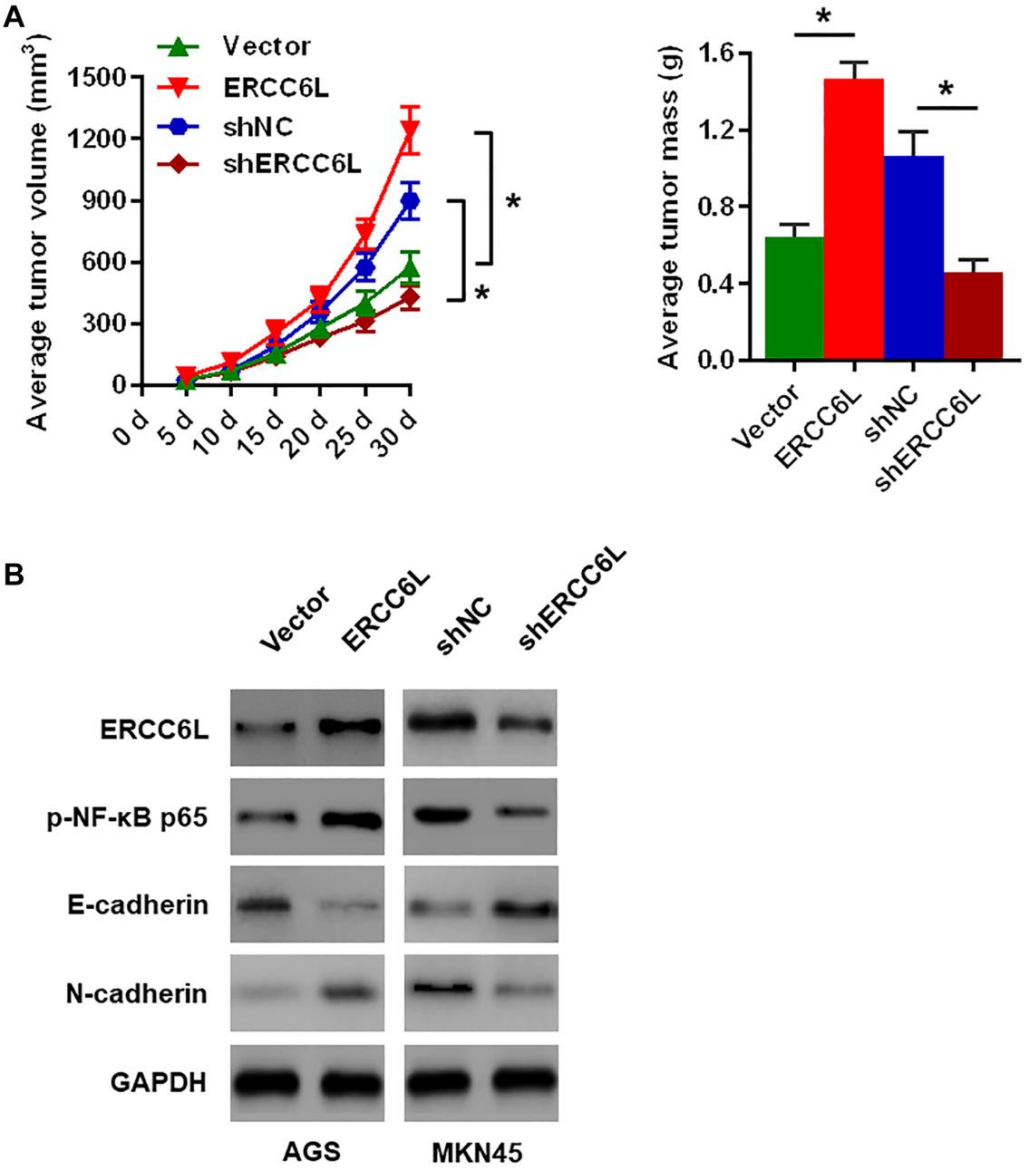
**The effects of ERCC6L overexpression or knockdown on GC cells growth *in vivo*.** (**A**) Representative images of the dissected xenograft tumors. Tumor volume was measured every 5 days. Tumors were weighed at the endpoint (30 days). (**B**) Western blot analysis of the levels of ERCC6L, p-NF-κB p65, E-cadherin and N-cadherin in the dissected xenograft tumors. (*n* = 6, each group). ^*^P < 0.05.

These results demonstrated ERCC6L overexpression in AGS cells markedly promoted cell proliferation on the basis of the volume and weight of the transplanted tumor (Figure 4A). The Western blot analysis of the xenograft tissues revealed that ERCC6L overexpression in AGS cells resulted in higher levels of Ki-67, p-NF-κB p65 and N-cadherin, and lower E-cadherin level (Figure 4B). Conversely, ERCC6L knockdown in MKN45 cells accomplished exactly the opposite (Figure 4A–4B).

**Figure 5:**
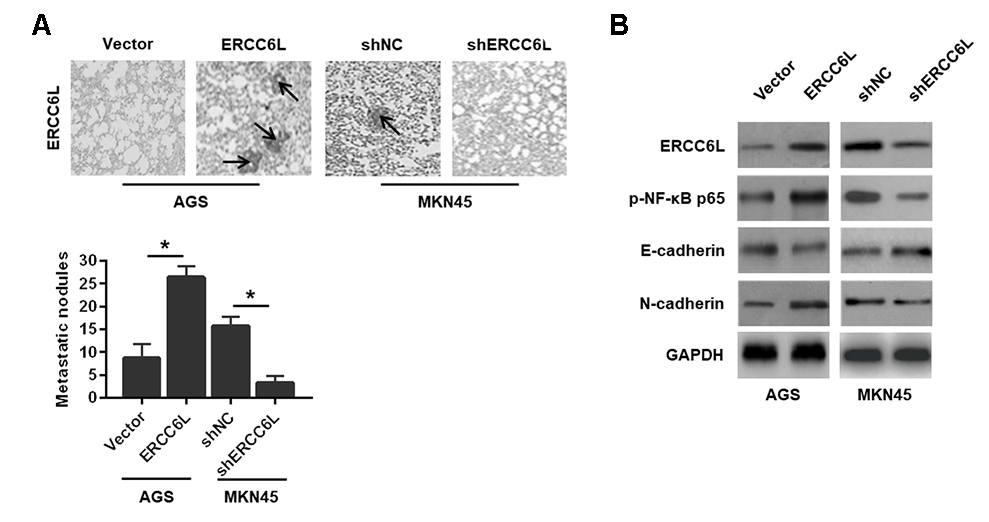
**The effects of ERCC6L overexpression or knockdown on GC cells metastasis *in vivo*.** (**A**) Representative images of the numbers of metastatic foci in lung of individual mouse. (**B**) Western blot analysis of the levels of ERCC6L, p-NF-κB p65, E-cadherin and N-cadherin in lung metastatic nodules. (*n* = 6, each group). ^*^P < 0.05.

In addition, the result revealed that ERCC6L overexpression in AGS cell promoted lung metastasis, as evidenced by the more pulmonary metastases (Figure 5A) and EMT reversal (Figure 5B). As expected, ERCC6L downregulation in MKN45 cells resulted in a significantly opposite effect on cell proliferation and metastasis *in vivo* (Figure 5A–5B).

Taken together, these findings verified the promotive role of ERCC6L in GC cell growth and metastasis.

### ERCC6L facilitates GC cell growth, migration and invasion via the NF-κB signaling pathway-induced EMT

**Figure 6:**
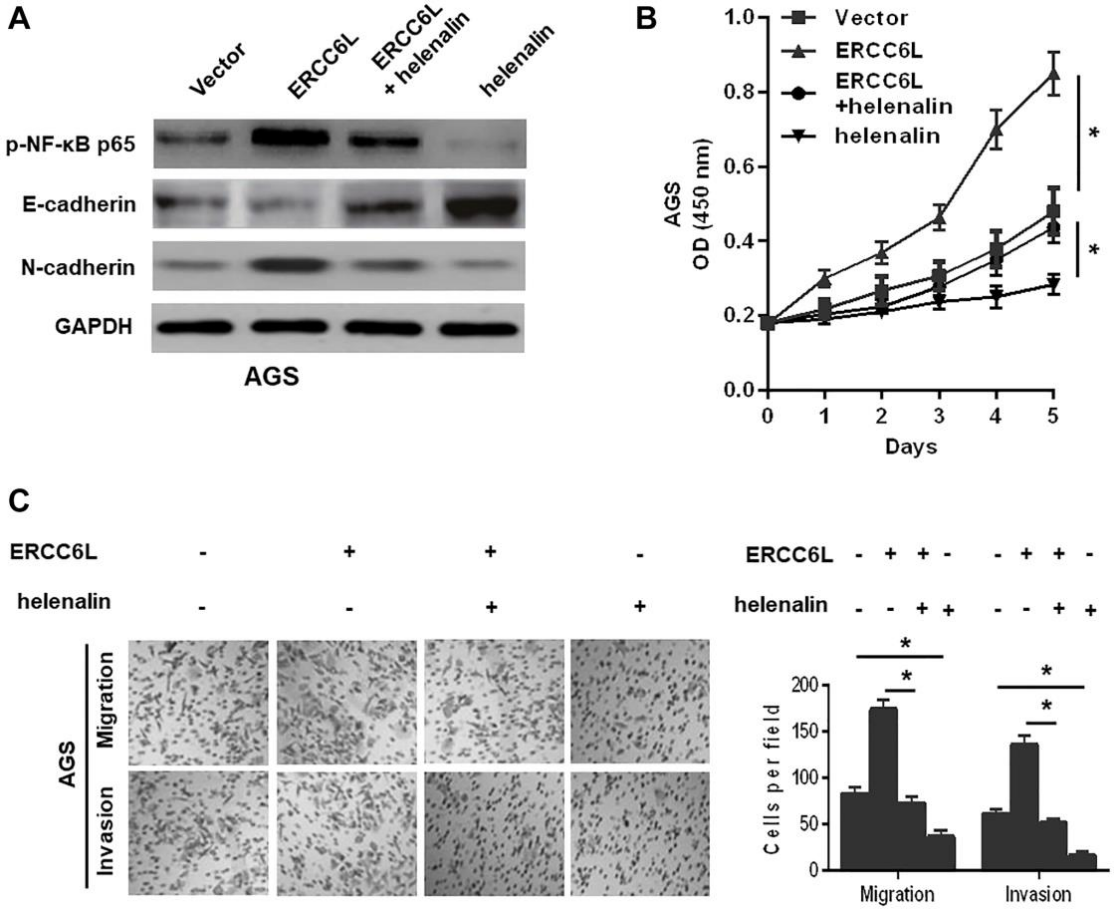
**NF-κB signal involved in ERCC6L-facilitated GC cell EMT, growth, migration, and invasion.** (**A**) Western blot analysis of the levels of p-NF-κB p65, E-cadherin and N-cadherin in modified AGS cells treated with helenalin (15 μM, NF-κB signaling inhibitor). (**B**) Determination of cell proliferation ability followed by the treatment with helenalin by CCK-8 assay. (**C**) Determination of cell migration and invasion abilities followed by the treatment with helenalin by transwell assay. ^*^P < 0.05.

The NF-κB signaling pathway is responsible for in tumor progression [9]. We reasoned that ERCC6L might promote EMT by activating NF-κB pathway in GC. We revealed that ERCC6L overexpression increased the levels of p-NF-κB p65 and N-cadherin; whereas, ERCC6L knockdown decreased their levels. To explore whether NF-κB signaling pathway involved in ERCC6L-induced cellular changes, helenalin, the inhibitor of NF-κB, was used. The results showed that helenalin could reverse EMT, decrease p-NF-κB p65 (Figure 6A) and suppress cell growth (Figure 6B), migration and invasion (Figure 6C) induced by ERCC6L. Altogether, these data demonstrated that NF-κB pathway-mediated EMT was involved in the ERCC6L-induced oncogenic function.

## DISCUSSION

GC remains a common and aggressive malignancy worldwide. Given that tumorigenesis and metastasis of GC is a complicated process and is affected by numerous genetic changes [10–11], it is urgently required to determine novel molecular targets and the potential mechanisms responsible for this process. Remarkably, recent reports have identified ERCC6L as a critical mediator in the malignant biological behavior of various cancers [7–8, 12]. For example, ERCC6L expression is increased in HCC tissue, which significantly correlates with poor clinical outcomes [7]. Furthermore, ERCC6L promotes tumor progression via the PI3K/AKT and NF-κB pathway in HCC [12]. In CRC, ERCC6L is significantly elevated in tumor tissues, and functions importantly in CRC cell growth, describing ERCC6L as a target for CRC [8]. Nevertheless, little is known about its function, especially in GC, and the molecular mechanisms of how ERCC6L influences GC progression and development.

Herein, we evaluated ERCC6L levels in GC tissues and GC cell. Clinical data showed that ERCC6L expression was positively associated with poor clinical outcomes. Survival analysis showed that GC patients with high ERCC6L expression had poor prognosis, suggesting that ERCC6L may be a valuable biomarker for GC. Given that ERCC6L correlates with unfavorable clinicopathological parameters in GC patients, we subsequently assessed the effect of ERCC6L on the malignant process of GC both. The result revealed that ERCC6L facilitated the growth, migration, invasion, and metastasis of GC cells. Mechanistically, we found that ERCC6L overexpression increased the expression of N-cadherin and decreased the expression of E-cadherin; while ERCC6L knockdown significantly reversed these events, suggesting that ERCC6L affected the malignant biological behavior of GC cells by modulating EMT.

As mentioned in the introduction, it is believed that EMT is a hallmark of cancer and a key part in tumor progression and metastasis in multiple types of malignancies, [5]. In recent years, more and more attention has been paid to EMT pathway in tumor therapy, and it is expected to become a target for the prevention of tumor development to some extent [5]. Herein, we identified important roles for the NF-κB pathway in the ERCC6L-induced EMT and aggressive behavior of GC cells. As mentioned earlier, NF-κB pathway has been considered as not only a critical element in cancer progression and metastasis but also an important modulator of EMT-related transcription factors [13–14]. Mechanistically, there was a dramatic increase in the phosphorylation of NF-κB p65 in GC ERCC6L-transfected cells, implying the activation of NF-κB pathway. Conversely, ERCC6L knockdown markedly inactivated the NF-κB pathway. Furthermore, to further ascertain the involvement of NF-κB signaling in ERCC6L-induced EMT, pretreatment of GC cells with helenalin (an NF-κB inhibitor) [15] resulted in blockade of the increase in ERCC6L-induced EMT, and cell growth, migration and invasion. Taking these factors together, we could assume that ERCC6L contributed to GC cell growth and metastasis through activation of NF-κB-induced EMT.

Nevertheless, it is worth further investigating the indirect or direct relationship between ERCC6L and NF-κB signal. As reported, ERCC6L could be involved in centromeric chromatin remodeling as a binding partner and substrate of PLK1, which is a key kinase in cell cycle progression [16]. Thus, it is of great importance to evaluate whether ERCC6L-induced EMT involves other genes or some signaling pathways in subsequent studies.

In conclusion, this study demonstrates that high ERCC6L expression significantly correlates with clinical proliferation and metastasis, and unfavourable prognosis in GC patients. Besides, our work identifies a critical role of ERCC6L in promoting the EMT process through NF-κB signaling pathway, which further facilitates GC cell growth, invasion, and metastasis, possibly providing a promising target to suppress GC progression and development.

## MATERIALS AND METHODS

### Tissue samples

100 pairs of tumor tissues were obtained from patients with GC undergoing radical resection at the Department of General Surgery of Taizhou People’s Hospital. The study was approved by the Ethics Committee of our Hospital. According to the Helsinki declaration, the written informed consent of all participants was obtained.

### Cell lines

GC cell lines (AGS, MKN28, SGC7901, BGC823, KATO-III, MGC803, and MKN45) andGES-1 were purchased from American Type Culture Collection (ATCC, USA). All cell lines were maintained in DMEM containing 10% serum at 37°C with 5% CO_2_.

### qRT-PCR

Total RNA was extracted from cells or GC tissues, and was reversely transcribed into cDNA using PrimeScript RT Reagent Kit (TaKaRa, Japan) according to the manufacturer’s instructions. All reactions were conducted using the SYBR Green assay kit (Takara, Japan). The mRNA level was evaluated according to the 2^-ΔΔCt^ method. Primers are summarized in Supplementary Table 1 [12].

### Western blot assay

Briefly, whole-cell or tissue lysates were prepared and equivalent amounts of proteins were transferred to PVDF membranes. The immune blots were blocked in 5% skimmed milk and then incubated with primary antibody and appropriate secondary antibody. The blots were visualized in accordance with instructions of an enhanced chemiluminescence kit. The primary antibody against ERCC6L was obtained from Abcam (Cambridge, UK). Primary antibodies against p-NF-κB p65, NF-κB p65, E-cadherin, and N-cadherin were purchased from CST (Danvers, MA, USA). [17].

### IHC and evaluation

In short, paraffin embedded sections are cut, deparaffined, rehydrated, and then heat treated for antigen recovery. Next, the slices were incubated with 3% hydrogen peroxide to inactivate endogenous peroxidase activity, and then incubated with 5% normal blocking serum. After that, the sections were treated with specific primary antibody overnight, then biotinylated secondary antibody (boster, Wuhan, China) and SABC solution. Finally, the sections were observed and imaged by light microscope.

IHC staining intensity (0, negative; 1, Weak; 2, Medium; 3, strong) and immunostaining area (0, 0–5%; 1, 6–25%; 2, 26–50%). If the total score is less than 3, it is negative. If the total score is more than 3, it is positive.

### Lentivirus infection

The commercial ERCC6L specific short hairpin RNA (shRNA) construction and overexpression construction were from genechem (Shanghai, China). Cells transfected with control shRNA or vector were used as control. According to the manufacturer’s instructions, Lipofectamine 2000 (Invitrogen) was used for cell transfection. The transfection efficiency was detected by Western blot.

### Cell proliferation assay

The cells were seeded in 96 well plates. Cell proliferation was detected by cell counting kit-8 (CCK-8) on day 0, 1, 2, 3, 4 and 5 after culture followed the manufacturer's instructions. The absorbance at 450 nm was detected and the cell growth rate was calculated.

### Colony formation assay

In short, the cells were cultured in 6-well plates for 14 days, then fixed in paraformaldehyde. Then, the cells were stained with crystal violet and counted under light microscope. The number of colonies (> 50 cells/colony) was calculated.

### EdU assay

In short, the cells were cultured with Edu for 2 h, fixed in paraformaldehyde, infiltrated into Triton X-100, and then stained with Apollo solution for 0.5 h. The cells were then stained with DAPI solution and observed under fluorescence microscope.

### Wound healing assay

In short, the cells were cultured into a monolayer. After that, the tip of the micropipette was used to form the wound space, and then the cell debris is removed. At the designated time points (0 h and 48 h), the wound images were taken to evaluate the remaining distance. The wound coverage rate was calculated as follows: wound closure rate = (0 h width–48 h width)/0 h width × 100%.

### Cell migration and invasion assays

Transwell chambers (Corning, New York, USA) and BD matrixtm basement membrane (BD Biosciences, San Jose, California, USA) were used to measure cell migration and invasion. These cells were maintained in serum-free medium and seeded in the upper cavity with or without matrix gel. The medium containing 20% fetal bovine serum was added to the bottom chamber. After incubation for 24 h, the cells were fixed in paraformaldehyde, stained with crystal violet solution, and counted in 5 random areas under the microscope [17].

### Animal studies

The cells were subcutaneously injected into nude mice (6-week-old, male) and the tumor volume was measured using the following formula: volume = (short diameter)^2^ × (Long diameter)/2 [17]. After 30 days, all mice were euthanized, the xenograft tissues were dissected, weighed and analyzed by Western blot. In addition, the cells were injected into nude mice via tail vein. Similarly, the lung of nude mice was excised 30 days after injection to count metastatic nodules, and then prepared for Western blot analysis [17]. The living animal experiment was approved by the ethics committee of the Fifth Affiliated Hospital of Nantong University.

### Statistical analysis

Data were presented as the mean ± standard deviation (SD), and were analyzed by Student’s *t*-test. All statistical tests were conducted using SPSS version 21.0 software. *P* < 0.05 was considered significant.

## Supplementary Materials

Supplementary Table 1
