# Supplementary material for: ERCC6L promotes cell growth and metastasis in gastric cancer through activating NF-κB signaling
Source: Aging (Albany NY). 2021 Aug 23;13(16):20218–28. doi: 10.18632/aging.203387 (PMC8436930; doi:10.18632/aging.203387)
Supplement: Supplementary Table 1 [file aging-13-203387-s001.pdf]

SUPPLEMENTARY TABLE

Supplementary Table 1. Primers designed for qRT-PCR.

Genes	Sequence (5′–3′)	
	**Forward**	**Reverse**
**ERCC6L**	AAGGATGAACGGACCAGAAAC	CTGTGAGGAGGAGGCGATTAC
**GAPDH**	AGGGGCCATCCACAGTCTTC	AGAAGGCTGGGGCTCATTTG
